# Effect of TiB_2_ Nanoparticle Content on the Microstructure and Mechanical Properties of TiB_2_/Mg-4Al-1.5Si Composites

**DOI:** 10.3390/ma16072852

**Published:** 2023-04-03

**Authors:** Jian Liu, Xiaogang Chen, Wuxiao Wang, Yu Zhao, Na He

**Affiliations:** 1 Faculty of Printing, Packaging Engineering and Digital Media Technology, Xi’an University of Technology, Xi’an 710054, China; 2 School of Material Science and Engineering, Xi’an University of Technology, Xi’an 710048, China

**Keywords:** Mg matrix composites, Mg_2_Si phases, TiB_2_, microstructure, mechanical properties

## Abstract

Coarse primary and eutectic Mg_2_Si phases were generally precipitated in Mg-Al-Si alloys during solidification at a low cooling rate, which tends to deteriorate the strength and ductility of magnesium alloys due to stress concentration. Different volume fractions of TiB_2_ nanoparticles (1%, 3%, and 5%) were added to an Mg-4Al-1.5Si alloy to refine the coarse Mg_2_Si phases based on a heterogeneous nucleation mechanism. The nanoparticles were incorporated and dispersed in the molten Mg alloys and by using semi-solid stirring followed by ultrasonic treatment (SSUT), and TiB_2_/Mg-4Al-1.5Si composites were obtained. The effect of TiB_2_ content on the microstructure and mechanical properties of the composites was studied. The results showed that the average size of primary Mg_2_Si phases and α-Mg grains decreased as the TiB_2_ content raised, the dendritic primary Mg_2_Si phases were refined into polygonal shapes with smaller sizes, and the refined primary Mg_2_Si phases were uniformly distributed in the alloys after adding 1 vol.% or 3 vol.% TiB_2_ nanoparticles. As the TiB_2_ content increased, the morphology of the eutectic Mg_2_Si phases was modified from coarse Chinese characters to short rod or fine dot shapes. Vickers hardness and yield strength of the composites reached a maximum (153 HV and 90.9 MPa, respectively) when TiB_2_ content was 5 vol.%, while the most superior ultimate tensile strength (142.4 MPa) and elongation (9.2%) were obtained when TiB_2_ content was 3 vol.%, which were improved by 173.2%, 31.5%, 69.8%, and 187.5%, respectively compared with the Mg-4Al-1.5Si alloys.

## 1. Introduction

Mg-Al-based alloys are the most used cast magnesium alloys due to their low cost, high strength, and good corrosion resistance [1]. However, their high-temperature strength and creep resistance deteriorate at a temperature above 120 °C due to the softening of the Mg_17_Al_12_ phase with low thermal stability (melting point is 437 °C) [2]. Mg-Al-Si series magnesium alloys not only possess excellent castability and low cost but also display good creep resistance at temperatures up to 150 °C due to the introduction of Mg_2_Si precipitate phases, which has a high melting point (1085 °C), high hardness (4600 HV), and high strength (1670 MPa) [3,4,5]. Fine precipitated Mg_2_Si phases can provide dislocation and grain boundary pinning [6]. However, at a low solidification rate, eutectic Mg_2_Si phases with Chinese character shapes generally precipitate from Mg-Al-Si alloys, and when the Si content exceeds 1.38 wt.%, coarse dendritic primary Mg_2_Si phases with sharp corners and edges may appear, which will cause stress concentration and become a crack source, thereby deteriorating the strength and ductility of the alloy [7]. Therefore, it is necessary to control the size, morphology, and distribution of the Mg_2_Si phases to improve the comprehensive mechanical properties of these alloys [8,9,10].

It has been reported that the addition of some ex situ or in situ ceramic particles to aluminum alloys containing a Mg_2_Si phase not only can refine and modify the Mg_2_Si phases based on heterogeneous nucleation mechanism but can also serve as a reinforcement, improving the mechanical properties. Li et al. [11] found that the addition of SiC nanoparticles can promote the heterogeneous nucleation of the eutectic Mg_2_Si phase to reduce its size in the Mg_2_Si/Al composite, and the shape was changed from a short strip to a dot shape. Zhao et al. [12] reported that, with the increase of the content of SiC particles from 5 wt.% to 10 wt.%, the morphologies of the primary Mg_2_Si in an Al-Si-Mg alloy remained unchanged, and the size of the primary Mg_2_Si phase decreased slightly. However, when SiC particle addition reached 15 wt.%, the primary Mg_2_Si particulates changed partially from polygonal to quadrangular with a decrease in size from 50 μm to 30 μm. The disregistry between the (0001) crystal plane of TiB_2_ and the (200) crystal plane of Mg_2_Si is 4.64% (less than 5% critical value); hence, TiB_2_ can theoretically be used as heterogeneous nuclei to refine the coarse Mg_2_Si phases [13,14], and this has been verified by our previous studies [15]. Xiao et al. [16] found that TiB_2_ particles can refine α-Mg grains in an AZ91 matrix, and as TiB_2_ particles increased, the size of α-Mg grains in the obtained TiB_2_/AZ91 composite decreased significantly. In addition, TiB_2_ is considered one of the ideal reinforcements for magnesium matrix composites due to its high hardness, high melting point, and strong chemical stability [17]. Sun et al. [18] added TiB_2_ microparticles to Mg-Li-Al alloys via stir casting and found that the main strengthening mechanism was grain refinement and the coefficient of thermal expansion mismatch. Compared with the micron-size particulate reinforcements or fibers, comparable or even superior mechanical properties can be achieved in the magnesium matrix nanocomposites by adding only a small number of nanoparticles [19,20]. The high volume fraction of nanoparticles can not only bring more heterogeneous nucleation sites for Mg_2_Si phases and α-Mg but can also improve the yield strength of the composites based on the Orowan mechanism [21]. However, it is a great challenge to disperse the high-volume fraction of nanoparticles in a metal melt due to their attractive van der Waals force [22]. Hence, it is necessary to determine an optimum value by investigating the effect of TiB_2_ content on the mechanical properties of the composites.

In the present work, TiB_2_ nanoparticles with different volume fractions were incorporated into an Mg-4Al-1.5Si alloy and dispersed using SSUT. Mg matrix composites hybrid reinforced by TiB_2_ nanoparticles, and in situ Mg_2_Si particles were obtained (TiB_2_/Mg-4Al-1.5Si composites), and the effects of TiB_2_ content on the microstructure and mechanical properties of the composites were systematically investigated.

## 2. Experimental Procedures

The Mg-4Al-1.5Si (wt.%) Mg alloy ingot was provided by Shanxi Yinguang Huasheng Magnesium Industry Co., Ltd., Wenxi, China. TiB_2_ nanoparticles with an average diameter of 50 nm were obtained from Shanghai Xiangtian Nanomaterials Co., Ltd., Shanghai, China. Three groups of TiB_2_/Mg-4Al-1.5Si composites containing 1 vol.%, 3 vol.%, and 5 vol.% TiB_2_ nanoparticles were fabricated by SSTU, respectively, with the following steps: a cylindrical crucible was heated to 700 °C by a resistance furnace, then the Mg alloy ingot was put into the crucible and kept for 50 min until the alloy was completely melted. Then, to incorporate these nanoparticles into the melt, the melt temperature was reduced to 635 °C in the semi-solid state, and the TiB_2_ nanoparticles, wrapped with aluminum foil and preheated at 635 °C were, fed into the melt. The melt containing TiB_2_ nanoparticles was mechanically stirred at 600 rpm for 5 min, during which the nanoparticles were sucked into the melt by axial flow and dispersed in the melt under shearing action [22]. The melt in the semi-solid state has a certain viscosity, which prevents the nanoparticles from floating or settling during the stirring [23]. After the mechanical stirring, the melt was rapidly heated to a liquid state at 720 °C again and subjected to ultrasonic treatment for 20 min (the ultrasonic frequency was 20 kHz, and the power was 1.8 kW), in which the nanoparticle aggregation was eliminated under a cavitation effect, and the nanoparticles were further dispersed under acoustic streaming [24]. The melting, stirring, and ultrasonic treatment was carried out under RJ-2 covering flux (32 wt.%~40 wt.%KCl-38 wt.%~46 wt.% MgCl_2_-3 wt.%~5 wt.% CaF_2_-5 wt.%~8 wt.% BaCl_2_) and in the atmosphere of argon gas to prevent melt oxidation and combustion. After slag removal, the melt was poured into a cylindrical mold of carbon steel. For convenience, the matrix alloy and the obtained composites with different volume fractions were denoted simply as M-AC, 1-COM, 3-COM, and 5-COM.

Specimens for microstructure observation and mechanical property tests were machined from the center of the cast ingot. After grinding and polishing, the metallographic specimens were etched with an acetic-picral reagent (5 mL acetic acid, 5 mL picric acid, 10 mL H_2_O, and 100 mL ethanol) for 10–15 s. The microstructures of the samples were observed by using OLYMPUS GX71 optical microscope (OM). Ten micrographs were taken from each the metallographic specimen as statistical analysis samples, and the equivalent diameters (*D*) of the primary Mg_2_Si phases in the alloy and the composites were determined using Image-Pro Plus 6.0 by Equation (1) [25]:

(1) $$D=2{\left(A/\pi \right)}^{1/2}$$

where *A* denotes the area of a single Mg_2_Si. The grain size of α-Mg was measured by the mean linear intercept method. Microstructure characterization was done using a JSM-6700F scanning electron microscope (SEM, JEOL, Tokyo, Japan) equipped with an energy-dispersive spectrum (EDS). Phase identification was carried out using an XRD-7000 X-ray diffractometer (Shimadzu, Kyoto, Japan). Tensile tests were conducted on an HT-2402 material testing machine at room temperature and 0.8 mm/min, the gauge dimension of the dog-bone tensile specimens is 15 mm × 5 mm × 2 mm, and at least three specimens for each material were tested to ensure the reproducibility of data. The hardness testing was taken on an HV-120 Vickers hardness tester with a load of 49 N and a dwell time of 15 s. To quantitatively evaluate the distribution of primary Mg_2_Si phases, ten metallographic photographs of different positions were selected from each material as analysis samples, the number of the primary Mg_2_Si phases was counted by Image-Pro Plus 6.0, and their distribution uniformity was evaluated by the relative standard deviation (RSD) method. The microstructures were divided into 2 × 4 sub-areas in the field of view region, and the amount of Mg_2_Si phases in each sub-area was counted. The standard deviation S was then determined by Equation (2):

(2) $$S=\sqrt{\frac{\sum_{i=1}^{n}{\left(x_{i}-\bar{x}\right)}^{2}}{n-1}}$$

where *n* denotes the number of sub-areas, $x_{i}$, and $\bar{x}$ represent the amount in the *i*th sub-area and the average amount of the primary Mg_2_Si phases, respectively. The smaller the *S* value, the more uniform the Mg_2_Si phases are distributed in the matrix.

## 3. Results and Discussion

### 3.1. Microstructures

**Figure 1:**
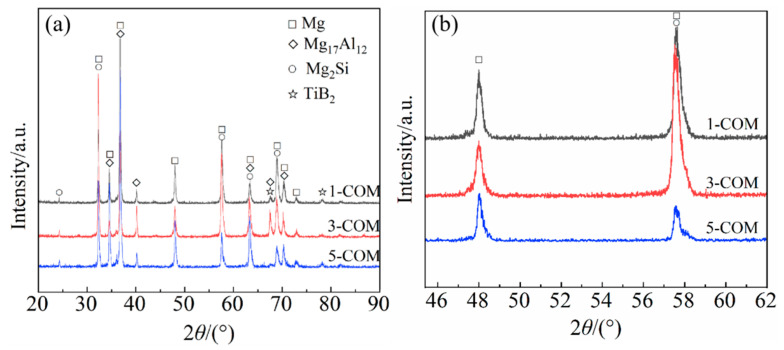
XRD patterns of TiB_2_/Mg-4Al-1.5Si composites: (**a**) 20° ≤ 2θ ≤ 90° and (**b**) 46° ≤ 2θ ≤ 62°.

Figure 1a shows the XRD diffraction patterns of the TiB_2_/Mg-4Al-1.5Si composites with different content of TiB_2_ nanoparticles (1 vol.%, 3 vol.%, and 5 vol.%). It can be seen from Figure 1a that the composites consist of α-Mg (JCPDS 04-0770), β-Mg_17_Al_12_ (JCPDS 73-1148), Mg_2_Si phases (JCPDS 34-0458), and TiB_2_ (JCPDS 35-0741). The diffraction peaks of TiB_2_ (JCPDS 35-0741) in the XRD diffraction patterns indicate that the TiB_2_ nanoparticles have been incorporated into the Mg alloys by SSUT, and no other new phases are found in the composites, indicating the TiB_2_ nanoparticles are thermally stable in the molten Mg alloys. In addition, the diffraction peaks of α-Mg shift towards the lower diffraction angles with the increase of TiB_2_ nanoparticle content, as shown in Figure 1b. According to Bragg’s equation [26],

(3) 2dsinθ=λ

where *λ* is the length of the incident wave, *d* is lattice spacing, and *θ* is the angle between the incident light and the crystal planes (Bragg diffraction angle). The lattice spacing (*d*) of the α-Mg increases as the diffraction angles (2θ) decrease to maintain the equation. The increase in the lattice spacing may be due to the heterogeneous nucleation of α-Mg on the TiB_2_ nanoparticles.

**Figure 2:**
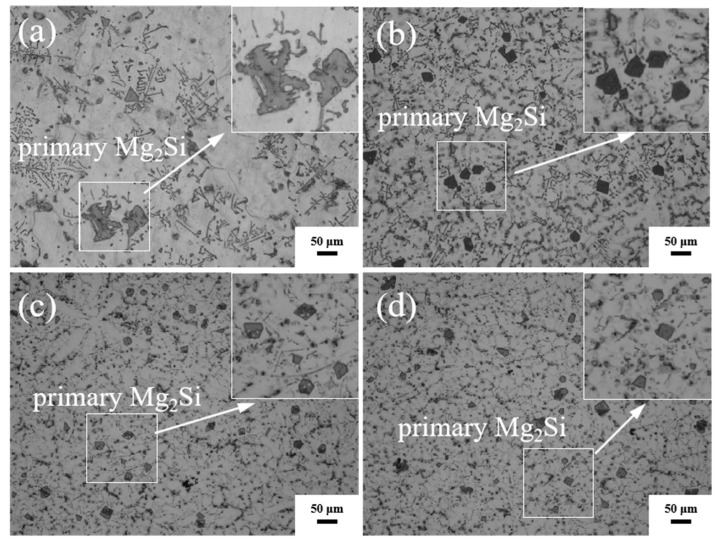
Optical micrograph of the alloys and composites, (**a**) M-AC, (**b**) 1-COM, (**c**) 3-COM, and (**d**) 5-COM showing the morphology and distribution of the primary Mg_2_Si. (Scale bar = 50 μm).

**Figure 3:**
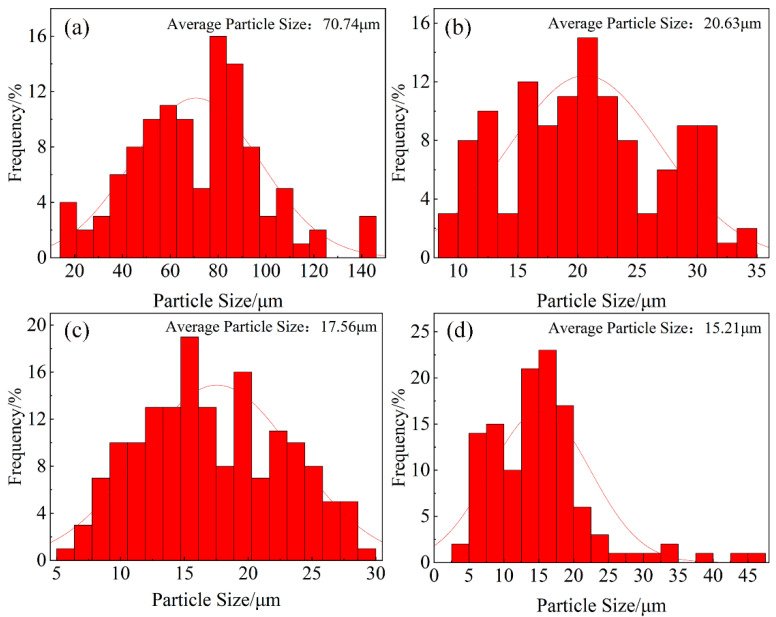
Size distributions of the primary Mg_2_Si phases in the alloys and the composites, (**a**) M-AC, (**b**) 1-COM, (**c**) 3-COM, and (**d**) 5-COM.

Figure 2 shows the morphology and distribution of the primary Mg_2_Si in the alloy and the composites. It can be found that the coarse dendritic primary Mg_2_Si phases are nearly transformed into fine polygons as the TiB_2_ content increases, as shown in Figure 2b–d. Figure 3 presents the size distributions of the primary Mg_2_Si phases in the alloys and the composites. The average diameter of the primary Mg_2_Si phases in the alloys is 70.74 μm, as shown in Figure 3a. The average diameter of the primary Mg_2_Si phases decreases to 20.63 μm after adding 1 vol.% TiB_2_ particles, and some particles are smaller than 10 μm in size, as shown in Figure 3b. When TiB_2_ content rises to 3 vol.%, the average diameter of the primary Mg_2_Si phases decreases to 17.56 μm, and the number of the primary Mg_2_Si phases smaller than 10 μm in size increases, as shown in Figure 3c. When TiB_2_ content reaches 5 vol.%, the average diameter of the primary Mg_2_Si decreases to 15.21 μm, and there exists a small amount of primary Mg_2_Si with a size less than 5 μm, while some primary Mg_2_Si phases with size greater than 40 μm appear again, as shown in Figure 3d. The continuous decrease in the average diameter of the primary Mg_2_Si phases with increasing TiB_2_ content indicates that the TiB_2_ nanoparticles significantly refined the primary Mg_2_Si phases.

**Figure 4:**
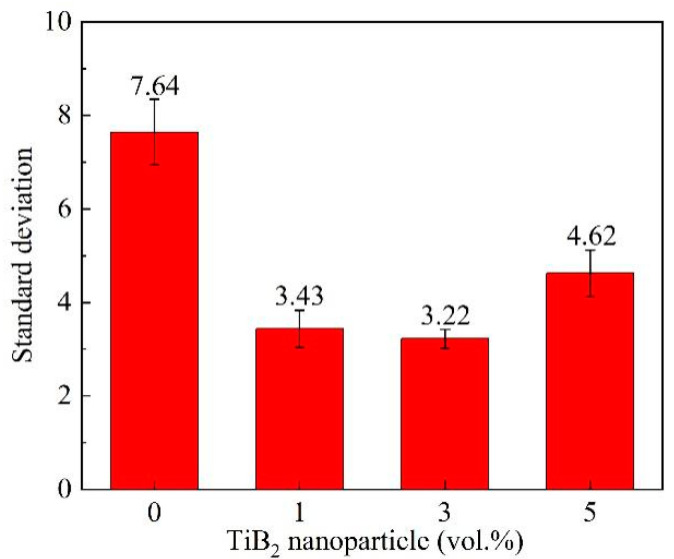
Standard deviation (*S*) of distribution uniformity of the primary Mg_2_Si phases.

Figure 4 shows the *S* value in the alloy and the composites. It can be seen from Figure 4 that, after adding 1 vol.% and 3 vol.% TiB_2_, the *S* value decreases from 7.64 to 3.43 and 3.22, respectively, while when TiB_2_ content increases to 5 vol.%, the *S* value reaches 4.62 abnormally, indicating that the addition of 1 vol.% or 3 vol.% TiB_2_ nanoparticles promoted the uniform precipitation of the primary Mg_2_Si phases during solidification, and the deteriorated distribution uniformity may be associated closely with the dispersion of the TiB_2_ nanoparticles in the 5-COM. It has been reported that when the addition of the TiB_2_ nanoparticles exceeds 3 vol.%, it is challenging to disperse the nanoparticles since they tend to form clusters and segregate around grain boundaries during solidification [27,28], and these clusters and segregations are hard to entrap using the primary Mg_2_Si phases. Hence, the distribution of the primary Mg_2_Si phases in the 5-COM is poorer than that in the 1-COM and 3-COM.

**Figure 5:**
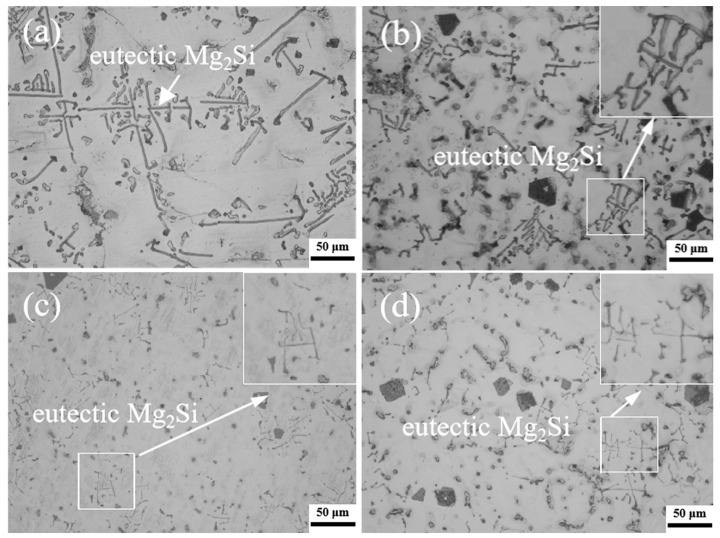
The morphologies of eutectic Mg_2_Si in the alloy and the composites (**a**) M-AC, (**b**) 1-COM, (**c**) 3-COM, and (**d**) 5-COM. (Scale bar = 50 μm).

Figure 5 illustrates the morphology of the eutectic Mg_2_Si phases in the alloy and the composites. The eutectic Mg_2_Si phases in the M-AC mainly exhibit Chinese characters or long-rod shapes, as shown in Figure 5a. After adding 1 vol.% TiB_2_ nanoparticles, a part of the eutectic Mg_2_Si phases is transformed to the short rod type, as shown in Figure 5b. When TiB_2_ content rises to 3 vol.%, the eutectic Mg_2_Si phases with Chinese characters and short rod shapes are refined remarkably, as shown in Figure 5c. When the TiB_2_ content reaches 5 vol.%, the eutectic Mg_2_Si phases mainly exhibit a thin rod shape and a dot shape, as shown in Figure 5d. It can be inferred from Figure 5 that the TiB_2_ nanoparticles also have a significant refining effect on the eutectic Mg_2_Si phases in the magnesium alloys, and the refining effect becomes increasingly remarkable as the TiB_2_ content escalates.

**Figure 6:**
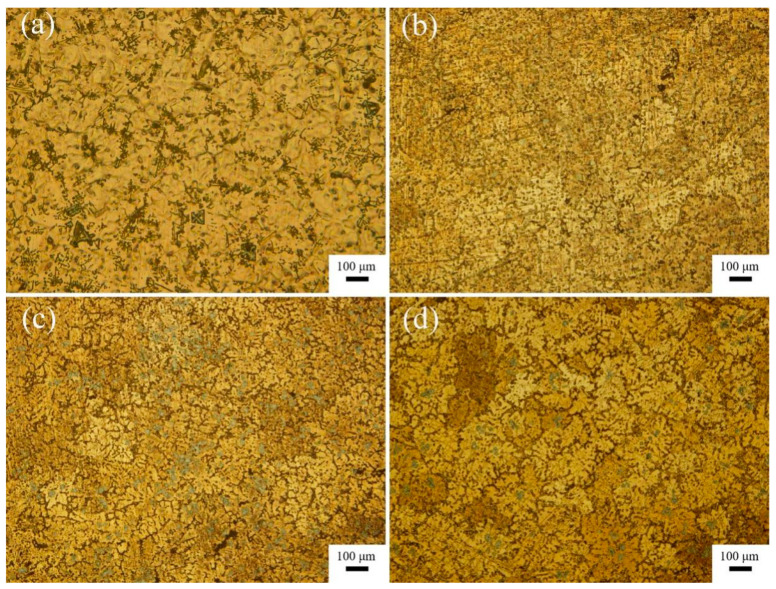
Metallographic photos of the alloys and composites showing the morphology of α-Mg grains: (**a**) M-AC, (**b**) 1-COM, (**c**) 3-COM, and (**d**) 5-COM.

**Figure 7:**
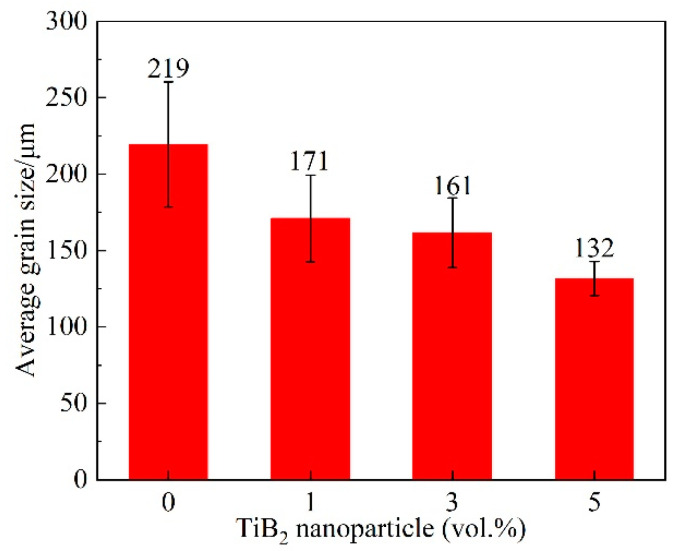
Variation of average grain size with vol.% of TiB_2_ nanoparticles.

Figure 6 presents the microstructure of the alloys and the composites showing the morphology of α-Mg grains. Figure 7 illustrates the average size of α-Mg grain in the alloy and the composites with different content of TiB_2_ nanoparticles. As shown in Figure 7, the average size of α-Mg grains in the alloys is about 219 μm. After adding 1 vol.% TiB_2_, the average size of α-Mg grains is about 171 μm. When TiB_2_ content is increased to 3 vol.%, the average size of α-Mg grains is about 161 μm. As the TiB_2_ content rises to 5 vol.%, the average size of α-Mg grains is about 132 μm. When the content of the TiB_2_ nanoparticles increases from 1 vol.% to 5 vol.%, the α-Mg grains are increasingly fine. The refinement of the α-Mg grains can be attributed to the following two aspects: first, the dispersed TiB_2_ nanoparticles acted as heterogeneous nucleation sites for α-Mg grains to refine α-Mg grain size [16]; second, the TiB_2_/Mg-4Al-1.5Si composite is an Mg matrix composite hybrid reinforced by TiB_2_ nanoparticles and micro-scale in situ Mg_2_Si phases, the TiB_2_ nanoparticles and the refined Mg_2_Si phases inhibited the growth of α-Mg grains. For the same volume fraction of reinforcement particles in metal matrix composites, smaller particles generally produce smaller grain sizes [29]. As the TiB_2_ content rises, the average size of the primary Mg_2_Si phases was reduced, and the fine primary Mg_2_Si phases exhibited a more and more obvious inhibition role on the α-Mg grains consequently.

### 3.2. Mechanical Properties

**Figure 8:**
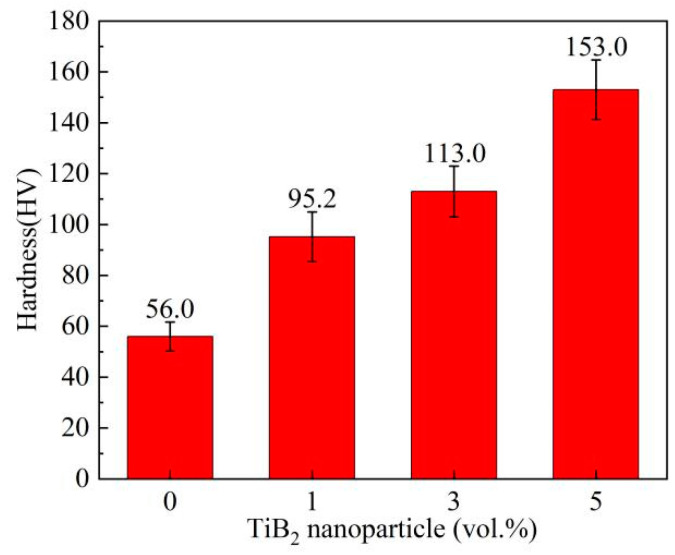
Variation of Vickers hardness with vol.% of TiB_2_ nanoparticles.

**Figure 9:**
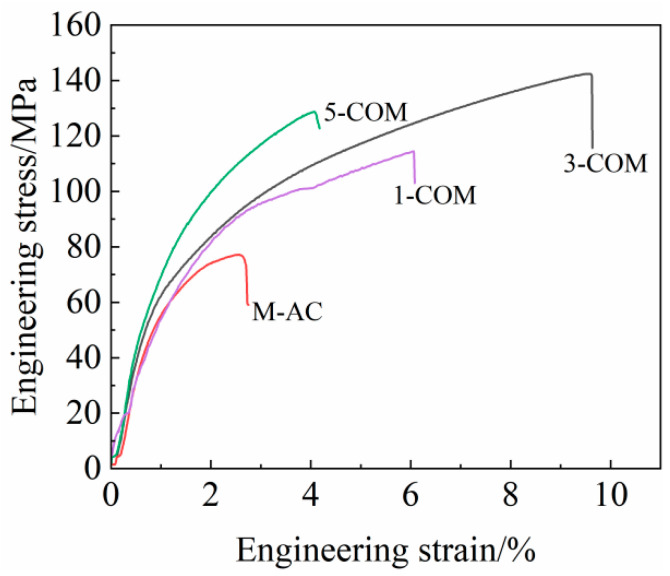
Typical tensile engineering stress-strain curves of the alloys and the composites.

Figure 8 shows the Vickers hardness of the alloys and the composites. It is seen that the hardness of the composites increases monotonically with the increase of TiB_2_ content. When TiB_2_ content is 5 vol.%, the hardness of the 5-COM reaches a maximum value of 153 HV, which is 173.2% greater than that of the matrix alloy. Figure 9 shows the engineering stress-strain curves of the alloys and the composites at room temperature, and the corresponding ultimate tensile strength (UTS), yield strength (YS), and elongation (EL) are listed in Table 1. UTS, YS, and EL of the TiB_2_/Mg-4Al-1.5Si composites with different TiB_2_ contents are higher than those of the matrix alloy. As the TiB_2_ content rises, YS increases monotonically, while UTS and EL increase at first and then decrease, reaching a maximum when TiB_2_ content is at 3 vol.%. The UTS, YS, and EL of the 3-COM are improved by 69.93%, 10.56%, and 187.5%, respectively, compared with the M-AC. When the TiB_2_ content rises to 5 vol.%, the YS of the 5-COM is improved by 31.5%, but the UTS and EL decrease slightly. Therefore, it can be concluded that the strength and ductility of the Mg-4Al-1.5Si alloy can be simultaneously enhanced by adding an appropriate amount of TiB_2_ nanoparticles, and desirable, comprehensive, mechanical properties can be obtained by adding 3 vol.% TiB_2_ nanoparticles. 

### 3.3. Strengthening and Toughening Mechanisms

The TiB_2_/Mg-4Al-1.5Si composites exhibit greatly improved hardness and strength, which can be attributed to the following aspects: firstly, the TiB_2_ nanoparticles and the refined Mg_2_Si phases are both desirable reinforcements with high hardness and high modulus, which have a strong pinning effect on grain boundary and dislocation [13]. Secondly, smaller secondary particles generally possess higher fracture stress; hence, the refined Mg_2_Si phases have a higher load-carrying capacity [30]. In addition, the average size of the α-Mg grain in the composites decreases with the addition of TiB_2_ nanoparticles, and the grain refinement improves the YS of the composites according to the Hall–Petch relationship [31].

**Figure 10:**
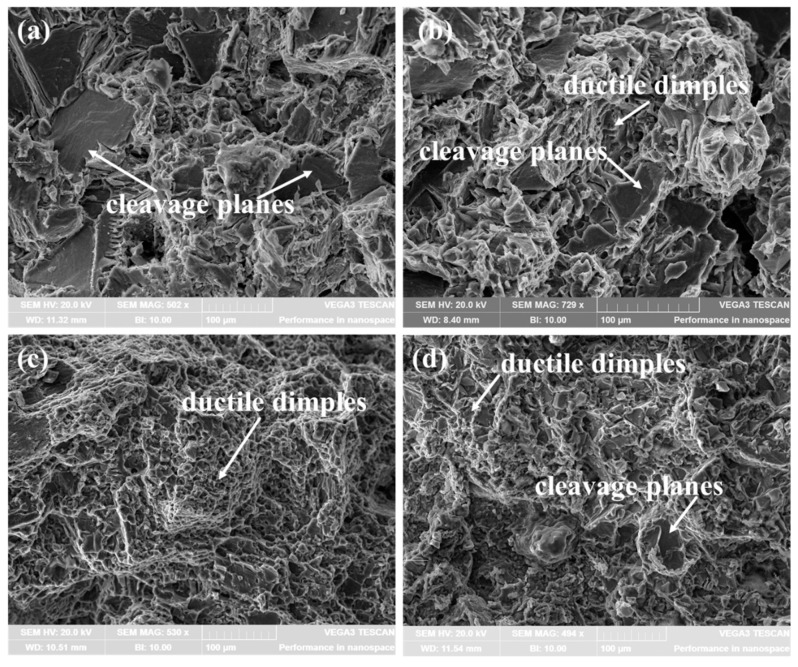
SEM images of fracture surfaces of the alloys and the composites: (**a**) M-AC, (**b**) 1-COM, (**c**) 3-COM, and (**d**) 5-COM.

The improvement of ductility of the composites is mainly due to the refinement and homogeneous dispersion of the Mg_2_Si phases. The primary Mg_2_Si phases and the eutectic Mg_2_Si phases in the M-AC exhibit a coarse, dendritic shape and a Chinese character shape, respectively, and the sharp tips and edges of the Mg_2_Si phases provide an easy path for crack propagation, and the fracture path moves preferentially through these regions. Therefore, the EL of the Mg-4Al-1.5Si alloy is only 3.2%, as shown in Table 1. Figure 10 presents the fracture surfaces of the alloy and the composites. The fracture surface of the alloys is characterized by smooth cleavage planes, suggesting that it belongs to a brittle fracture mode (see Figure 10a). After adding 1 vol.% TiB_2_ nanoparticles, the stress concentration level is decreased, and microcrack initiation is delayed due to the refinement and uniform distribution of the Mg_2_Si phases, and the EL of 1-COM consequently increased to 5.1% (Table 1). As shown in Figure 10b, the area of the cleavage planes decreased in the fracture surface of the 1-COM, and a small number of dimples appeared on the fracture surfaces, which means it was a quasi-cleavage fracture mode. When the TiB_2_ content rose to 3 vol.%, the Mg_2_Si phases were further refined, and the distribution of the primary Mg_2_Si phases was more uniform, which increased the compatible deformation capability of the composites, the EL of 3-COM consequently increased to 9.2% (Table 1). The fracture surfaces of the 3-COM were almost covered with a large number of small dimples, indicating the dominant fracture behavior is a ductile fracture (see Figure 10c). When the TiB_2_ content reached 5 vol.%, due to the relatively lower distribution uniformity of the primary Mg_2_Si phases and the existence of large primary Mg_2_Si phases in the 5-COM, the EL of 5-COM was only 3.4% (Table 1). Dimples and cleavage planes coexist in the fracture surface of the 5-COM, indicating that the fracture mode changed from ductile fractures to quasi-cleavage fractures again (Figure 10d).

## 4. Conclusions

The different volume fractions of TiB_2_ nanoparticles (1%, 3%, and 5%) were incorporated into an Mg-4Al-1.5Si alloy by SSUT. Through microstructure observation and mechanical property testing, three main conclusions were drawn as follows:

(1) The TiB_2_ nanoparticles not only refined the α-Mg grains but also refined the Mg_2_Si phases in the composites. As the TiB_2_ content increased, the average size of the α-Mg grains and the primary Mg_2_Si phases decreased remarkably.
(2) When the content of the TiB_2_ nanoparticles was 1 vol.% or 3 vol.%, the primary Mg_2_Si phases were distributed uniformly in the Mg alloys, but the distribution uniformity decreased slightly when the TiB_2_ content raised to 5 vol.%.
(3) As the TiB_2_ content raised, the Vickers hardness and YS of the composites increased monotonically; the UTS and EL initially increased, followed by a decrease, reaching a maximum value when the TiB_2_ content was 3 vol.%, achieving a synergistic improvement in strength and ductility.

## Figures and Tables

**Table 1:** Mechanical properties of the alloys and the composites.

Materials	Hardness/HV	UTS/MPa	YS/MPa	EL/%
M-AC	56.0 ± 5.9	83.8 ± 14.1	69.1 ± 5.8	3.2 ± 0.8
1-COM	95.2 ± 9.7	108.8 ± 15.9	74.3 ± 11.7	5.1 ± 0.3
3-COM	113.0 ± 10.0	142.4 ± 11.4	76.4 ± 10.2	9.2 ± 1.1
5-COM	153.0 ± 11.7	125.3 ± 9.7	90.9 ± 9.3	3.4 ± 1.4

## Data Availability

Not applicable.
